# Digital Empathy in Aging Societies: Co-constructing the Concept with Students across Finland and China

**DOI:** 10.1093/geroni/igaf122.3570

**Published:** 2025-12-31

**Authors:** Yaru Li, Honglin Chen, Yanan Jiang, Ning An

**Affiliations:** University of Eastern Finland, Kuopio, Pohjois-Savo, Finland; University of Eastern Finland, Kuopio, Pohjois-Savo, Finland; University of Wisconsin-Madison, Madison, Wisconsin, United States; Hefei University of Technology, Hefei, Anhui, China (People’s Republic)

## Abstract

As services and education digitize in aging societies—accelerated by rapid advances in AI—cultivating “digital empathy,” the cognitive, affective, and communicative expression of empathy via digital technologies, is essential to support older adults’ inclusion. We co-constructed this concept with social work students in two contexts (62 master’s students in fall 2024 at the University of Eastern Finland, Finland; 52 undergraduate students in spring 2025 at Anhui Institute of Medicine, China) and identified actionable behaviors and design cues. In each setting, students received a brief empathy primer, viewed a short video of older adults encountering digital barriers, then wrote their own definition of “digital empathy.” Responses were analyzed using bilingual reflexive thematic analysis with a hybrid codebook and back-translation. Across sites, students converged on three dimensions: (1) communicative presence and empathic signaling online (e.g., appropriate camera use, timely responses, check-ins, supportive reactions); (2) perspective-taking with respectful, clear, plain-language communication; and (3) inclusive support and empathic design for older adults, including non-digital alternatives, patient coaching without shaming, and advocacy for accessibility/usability. Contextual emphases differed: Finnish definitions highlighted communicative presence and leadership-driven psychological safety in hybrid teams, whereas Chinese definitions foregrounded perspective-taking, practical assistance for older adults, and empathic, personalized design (with some invoking AI-enabled support). Findings inform curricula and service norms (e.g., check-in rituals, consent-based communication, plain language) and translate into older-adult-inclusive design prompts (offer access alternatives; prioritize accessibility/usability; normalize supportive reactions).

